# Cichoriin, a Biocoumarin, Mitigates Oxidative Stress and Associated Adverse Dysfunctions on High-Fat Diet-Induced Obesity in Rats

**DOI:** 10.3390/life12111731

**Published:** 2022-10-28

**Authors:** Hany Ezzat Khalil, Miada F. Abdelwahab, Hairul-Islam Mohamed Ibrahim, Khalid A. AlYahya, Abdullah Abdulhamid Altaweel, Abdullah Jalal Alasoom, Hussein Ali Burshed, Marwan Mohamed Alshawush, Shaimaa Waz

**Affiliations:** 1 Department of Pharmaceutical Sciences, College of Clinical Pharmacy, King Faisal University, Al-Ahsa 31982, Saudi Arabia; 2 Department of Pharmacognosy, Faculty of Pharmacy, Minia University, Minia 61519, Egypt; 3 Department of Biological Sciences, College of Science, King Faisal University, Al-Ahsa 31982, Saudi Arabia; 4 Pondicherry Centre for Biological Science and Educational Trust, Puducherry 605004, India; 5 Department of Surgery, College of Medicine, King Faisal University, Al-Ahsa 36363, Saudi Arabia; 6 Department of Biochemistry, Faculty of Pharmacy, Minia University, El-Minia 61511, Egypt

**Keywords:** cichoriin, obesity, high-fat diet, ALT, urea, creatinine, MDA, PPAR-γ

## Abstract

Metabolic dysfunctions linked to obesity carry the risk of co-morbidities such as diabetes, hepatorenal, and cardiovascular diseases. Coumarins are believed to display several biological effects on diverse adverse health conditions. This study was conducted to uncover the impact of cichoriin on high-fat diet (HFD)-induced obese rats. Methods: Obesity was induced in twenty rats by exposure to an HFD for six weeks. The rats were randomly divided into five groups; group I comprised five healthy rats and was considered the control one. On the other hand, the HFD-induced rats were divided into the following (five per each group): group II (the HFD group), groups III (cichoriin 50 mg/kg) and IV (cichoriin 100 mg/kg) as the treatment groups, and group V received atorvastatin (10 mg/kg) (as a standard). Triglycerides (TG), total cholesterol (TC), high-density lipoprotein cholesterol (HDL-C), low-density lipoprotein cholesterol (LDL-C), alanine transaminase (ALT), aspartate transaminase (AST), creatine kinase MB (CK-MB), urea, creatinine, the hepatic and renal malondialdehyde (MDA) as well as reduced glutathione (GSH) levels were assessed. Histopathological analysis of the heart, kidney, and liver tissues was investigated. mRNA and protein expressions of the peroxisome proliferator-activated receptor gamma (PPAR-γ) were estimated. Results: The administration of cichoriin alleviated HFD-induced metabolic dysfunctions and improved the histopathological characteristics of the heart, kidney, and liver. Additionally, the treatment improved the lipid profile and hepatic and renal functions, as well as the oxidative balance state. Cichoriin demonstrated an upregulation of the mRNA and protein expressions of PPAR-γ. Taken together, these findings are the first report on the beneficial role of cichoriin in alleviating adverse metabolic effects in HFD-induced obesity and adapting it into an innovative obesity management strategy.

## 1. Introduction

Obesity is a pathological condition of immoderate fat accumulation in the body due to a long-term imbalance between energy intake and expenditure [1]. Besides endocrine disorders, genetic variations as well as medicinal and nutritional changes can also lead to the progression of obesity [2]. According to the World Health Organization, nearly one in three adults is overweight, and more than one in eleven adults has severe obesity [3]. In the past few years, the prevalence of obesity has been increasing and is becoming a global epidemic [4]. Obese patients are susceptible to serious health problems because obesity is closely associated with metabolic diseases involving dyslipidemia, type 2 diabetes, and fatty liver, in addition to renal and cardiovascular dysfunction and, therefore, it poses a huge socio-economic burden [1,5,6]. Obesity also has a considerable impact on the patient’s social and psychological life, leading to the onset of depression [7]. Furthermore, obesity can induce oxidative stress through several mechanisms comprising oxidative phosphorylation, superoxide generation, and glyceraldehyde auto-oxidation [8]. Consequently, the antioxidant capacity is reduced, more pro-inflammatory cytokines are produced, and the cell structures are damaged, triggering cancer initiation and progression [3,9,10].

Currently, many therapeutic strategies are available for the treatment and/or prevention of obesity including lifestyle, nutrition education, physical exercises, and pharmacotherapy [11,12]. However, it is reported that conventional weight management through behavior modifications does not usually produce pronounced results [13]. Moreover, the potentially harmful adverse effects of most of the approved anti-obesity drugs limit their use [14,15,16]. On the other hand, weight-loss surgeries, which are recommended for patients with severe obesity, can provoke medical complications or recurrent operations [17]. As a consequence, alternative sources of safe, efficacious, and cost-effective weight reduction drugs are being explored [18,19]. Many phytogenic herbal products have demonstrated prominent anti-obesity properties through various mechanisms [1,3,20].

Coumarins are phenolic compounds belonging to the benzopyrone family. They can be naturally isolated from plants, bacteria, and fungi, and their synthetic analogs can be readily obtained in the laboratory [21,22]. The broad pharmacological activities of coumarins have attracted researchers for decades and have been reviewed in many reports [23,24,25]. Coumarins and their derivatives showed important therapeutic effects implicated in vasodilation [26], antioxidant properties [27], anti-inflammatory [28], anti-cancer [29], lipid-lowering [30], anti-diabetic [31] and anti-obesity [32]. These promising findings encouraged us to evaluate the anti-obesity activity of the coumarin glucoside, namely cichoriin (aesculetin 7-glucoside).

This study aimed to assess the in vivo anti-obesity activity of cichoriin. Accordingly, histopathological observations of the heart, liver, and kidneys were carried out, in addition to the investigation of the governing molecular mechanism.

## 2. Materials and Methods

### 2.1. Animals

Male albino Wistar rats were supplied from the National Research Center (Giza, Egypt) at a weight of 100–150 g and maintained on a 12:12-h light–dark cycle, with free access to a normal pellet diet and water. All the animals were habituated to laboratory conditions for one week before the experiment. The study protocol was approved by the Commission on the Ethics of Scientific Research with the approval number ES26/2020 at the Faculty of Pharmacy, Minia University, Egypt.

### 2.2. Induction of Obesity

The rats were fed a self-made high-fat diet (HFD) which provided 58% fat, 25% protein, and 17% carbohydrates as a percentage of total kcal. The composition of the HFD was prepared according to other previous studies [33,34], as shown in Table 1. The HFD was supplied to the rats for six weeks before starting the treatment protocol and continued during the treatment period.

### 2.3. Design of Study

A total of twenty-five rats were randomized into five groups (each group consisting of five animals). Group I: the control group fed on a normal pellet diet and 1% carboxy methyl cellulose (CMC) orally as a vehicle. Group II: the obesity group fed on the HFD and 1% CMC orally. Group III: the treatment group fed on the HFD and received cichoriin (Sigma-Aldrich, St. Louis, MO, USA) at 50 mg/kg/day, orally and dissolved in 1% CMC, for four weeks. Group IV: the treatment group fed on the HFD and received cichoriin at 100 mg/kg/day, orally and dissolved in 1% CMC, for four weeks. The selection of the doses was based on our preliminary study and a previous study on a similar coumarin compound [35]. Group V: the standard group fed on the HFD and received atorvastatin (Sigma-Aldrich, St. Louis, MO, USA) at 10 mg/kg, orally [36] and dissolved in 1% CMC, for four weeks. The weights of the rats were measured in grams (g) every two weeks and before scarification. Animal heights were measured from the nose to the anus, and the body mass index (BMI) was estimated by using the formula [37]: BMI = [Weight (g)/Height (cm^2^)].

The body-weight gain was assessed from the difference between final body weight and initial body weight.

### 2.4. Collection of Serum and Organ Samples

At the end of the study, the rats were sacrificed under anesthesia after overnight fasting. Blood samples were collected in centrifuge tubes and kept for 30 min, then the serum was separated by centrifugation at 3000 rpm for 15 min. The liver, kidneys, heart, and visceral fat were excised and weighed. Parts of each of the excised tissues were fixed in 10% buffered formalin and the other parts were stored at −20 °C.

### 2.5. Assessment of Biochemical Parameters

The serum triglycerides (TG) [38], total cholesterol (TC) [39], high-density lipoprotein cholesterol (HDL-C), and low-density lipoprotein cholesterol (LDL-C) [40] were evaluated using kits (Biodiagnostic, Giza, Egypt). The alanine transaminase (ALT) and aspartate transaminase (AST), as well as the urea and creatinine concentrations, were determined in serum according to the manufacturer’s protocols (Biomed, Cairo, Egypt). The creatine kinase MB (CK-MB) concentration was measured in serum by the kinetic UV method following the protocol of the commercial kit (Spectrum Diagnostic, Cairo, Egypt) [41].

### 2.6. Measurement of Antioxidant and Oxidative Stress Parameters

Liver and kidney homogenates (1:5 *w*/*v*) were prepared in z 0.1 mM PBS buffer (pH 7.4), and the supernatants after centrifugation were used for the estimation of hepatic and renal lipid peroxidation (malondialdehyde (MDA) level) and reduced glutathione (GSH). The levels of MDA, as a marker for lipid peroxidation, were determined chemically as thiobarbituric acid reactive substances (TBARS) according to the prescribed method by Khalil et al. [42] and expressed as nmol of TBARS/g tissue. The GSH was assessed following the procedures of the manufacturer (Biodiagnostic, Giza, Egypt) and expressed as mg/g tissue.

### 2.7. Histopathological Examination

Briefly, the cardiac, renal, and hepatic tissues were sliced to 3–4 mm thick, fixed in 10% neutral buffered formalin, dehydrated in graded concentrations of ethanol, cleared in xylene, and embedded in paraffin. The paraffin blocks were sectioned with a microtome at (4–6μm) thickness and dyed with Hematoxylin and Eosin (H&E) stain to study the general tissue structure [43]. The H&E-stained sections were examined using a Leica microscope (Leica Microsystems, Heerbrugg, Switzerland).

### 2.8. RT-PCR of PPAR-γ in Adipose Tissue

A nucleic acid extraction kit (Nucleospin, Macherey-Nagel GmbH & Co. Düren, Germany) was used for the extraction of the total RNA from homogenized adipose tissues. Then, the quantification and purity of the RNA samples were measured spectrophotometrically. RNA was used for the synthesis of complementary DNA (cDNA) followed by RT-RCR according to the manufacturer’s instructions for the kit (Bioline, London, UK). The primers sequence for the peroxisome proliferator-activated receptor gamma (PPAR-γ) gene was forward 5′CGAGTGCCGAGTCTGTGGGGATAA3′ and reverse 5′ATGGTGATTTGTCTGTTGTCTTTC3′, and the glyceraldehyde 3-phosphate dehydrogenase (GAPDH) housekeeping gene was forward 5′CCTCGTCTCATAGACAAGATGGT3′ and reverse 5′GGGTAGAGTCATACTGGAACATG3′. The prepared reaction mixtures were applied in a StepOne instrument (Step One Applied Biosystem, Foster City, CA, USA). The thermal profile cycling was as follows: 10 min at 45 °C for reverse transcription and 2 min at 95 °C for polymerase activation, followed by 40 cycles of 5 s at 95 °C, 10 s at 60 °C, and 5 s at 72 °C for the amplification step. Relative expression was determined by the Comparative Cycle threshold (2-∆∆Ct) Method and normalized to the GAPDH gene [42].

### 2.9. Western Blot Analysis of PPAR-γ Protein Expression in Adipose Tissue

The mouse monoclonal PPAR-γ antibody (Santa Cruz Biotechnology, CA, USA) was used. Briefly, the adipose tissue was homogenized, and then the protein was extracted and quantified by the Bradford method. Twenty μg of protein was denaturated by 5 min of boiling with an equal volume of a 2× Laemmli sample buffer containing 4% SDS, 10% 2-mercaptoethanol, 20% glycerol, 0.004% bromophenol blue, and 0.125 M Tris HCl, with a pH of 6.8. Samples were loaded for 10% SDS–PAGE for separation of the protein. The separated protein bands were electro-blotted on a PVDF membrane. The membrane was blocked in a Tris-buffered saline-Tween 20 (TBS-T) buffer with 3% bovine serum albumin. Incubation with the primary antibody was conducted overnight at 4 °C, followed by 3 times washing with TBS-T. Incubation with the HRP-conjugated secondary antibody was conducted for 1 h at room temperature, followed by 3 times washing with TBS-T. The chemiluminescent substrate (Bio-Rad, CA, USA) was used for the visualization of the protein bands. The band intensity of the target protein was analyzed against β-actin (Cell Signaling Technology, Beverly, MA, USA) on Image J software [44].

### 2.10. Statistical Analysis

Results were presented as means ± SEM. A one-way analysis of variance followed by a Tukey–Kramer post hoc test was used to compare the groups. Statistical significance was set as *p* < 0.05. GraphPad prism (version 7) was used for the statistical analysis.

## 3. Results

### 3.1. Effect of Cichoriin on Body Weight, BMI, and Organ Weight of Obese Rats

As summarized in Table 2, the rats fed with the HFD significantly increased in body-weight gain and BMI when compared to that of the control rats. Also, the rats’ organs and visceral fat weights were significantly increased in the HFD rats. Interestingly, cichoriin administration, especially a high dose of 100 mg/kg, could significantly decrease all of the aforementioned weight parameters compared to that of the HFD group. There was a non-significant difference between the cichoriin (100 mg/kg) and atorvastatin groups.

### 3.2. Effect of Cichoriin on Lipid Profile of Obese Rats

The HFD caused a significant elevation of the serum TC, TG, LDL-C levels, and LDL-C/HDL-C ratio with a significant decrease in HDL-C as compared to that of the control group. However, the administration of cichoriin with the HFD could significantly improve the lipid profile parameters and returned them to that of the control rats. A high dose of cichoriin (100 mg/kg) showed better improvement in the lipid profile than the low dose (50 mg/kg) and was equivalent to the effect of atorvastatin administration (Table 3).

### 3.3. Effect of Cichoriin on Biochemical Parameters of Obese Rats

**Figure 1:**
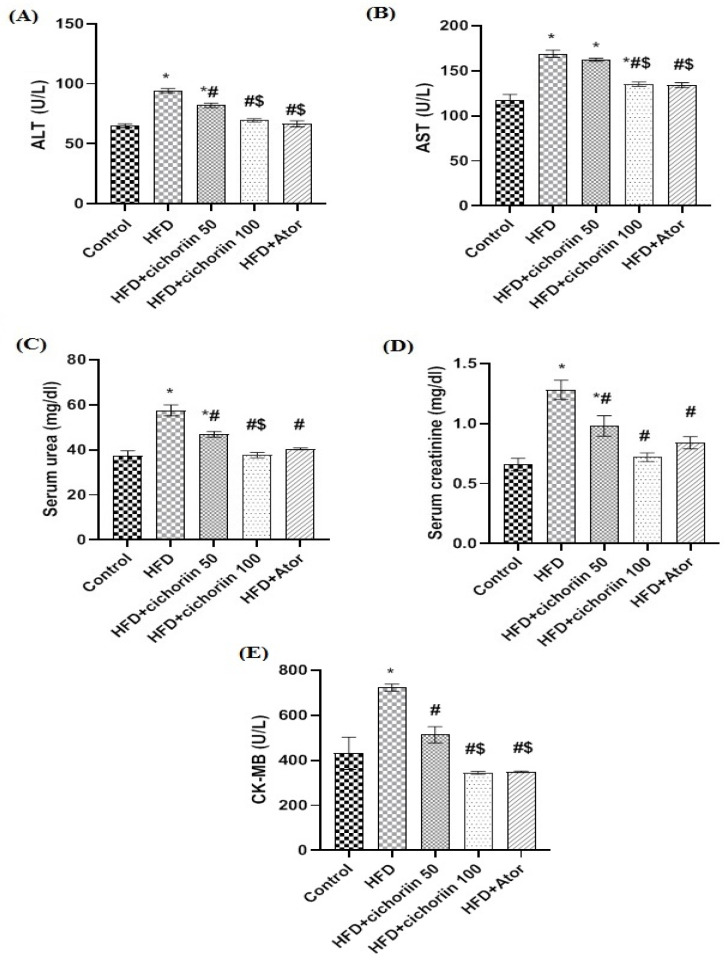
Effect of cichoriin on serum biochemical parameters of obese rats. ALT (**A**), AST (**B**), urea (**C**), creatinine (**D**), and CK-MB (**E**). Data are presented as means ± SEM (n = 5). *, ^#^, and ^$^ indicate significant difference from the (control), (HFD), and (HFD + cichorin 50 mg/kg) groups, respectively, at (*p* < 0.05). High-fat diet (HFD); atorvastatin (Ator); alanine transaminase (ALT); aspartate transaminase (AST); creatine kinase MB (CK-MB).

The liver function enzymes, ALT and AST (Figure 1A,B, respectively), as well as the renal function tests and the urea and creatinine (Figure 1C,D, respectively), were significantly elevated in the serum of the HFD group compared to the control. Additionally, the HFD significantly increased the cardiac isoenzyme and the CK-MB (Figure 1E) serum level compared to the control. Although the low dose of cichorin (50 mg/kg) could not significantly normalize the elevated serum levels of the hepatic and renal functions, the high dose (100 mg/kg) showed a significant decrease in the affected parameters compared to the HFD group.

### 3.4. Effect of Cichoriin on Hepatic and Renal Oxidative Stress Markers of Obese Rats

The rats fed with the HFD exhibited an imbalance of the oxidative state as there was a significant increase in MDA, the lipid peroxidation marker, with a decrease of the antioxidant GSH level in both the hepatic and renal tissues compared to that of the control rats. Alternatively, both doses of cichoriin (50 and 100 mg/kg) significantly returned the oxidative balance compared to the HFD group but was still significant from the control. The high dose of the cichoriin and atorvastatin groups showed a significant improvement in the oxidative parameters than the low cichoriin dose (Table 4).

### 3.5. Histopathological Analysis

#### 3.5.1. Examination of Heart Tissue

**Figure 2:**
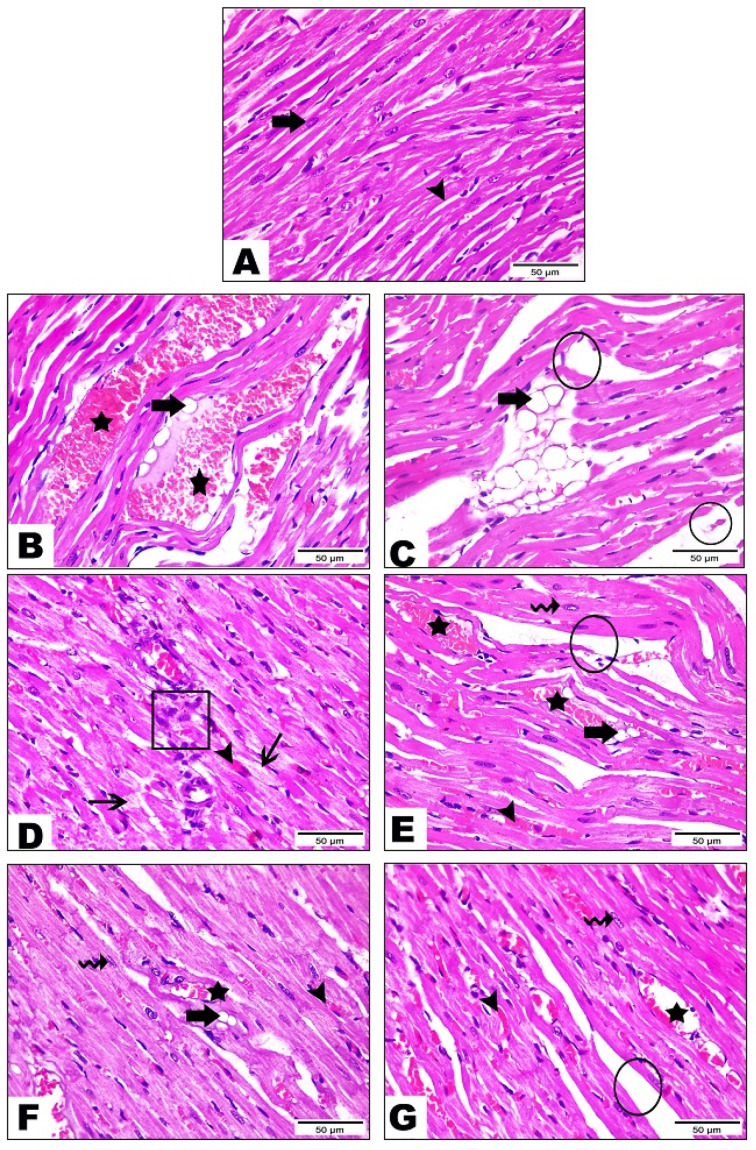
Photomicrograph sections of the control and HFD heart tissue of rats with or without treatments. Group I (control) (**A**) showed elongated, branched, and cross-striated cardiomyocytes with large oval central nuclei (thick arrow) and narrow slit-like interstices (arrowhead), group II (HFD) (**B**–**D**) showed vascular congestion (star), hemorrhage (arrowhead), excessive lipid deposition (thick arrow), distinct interstitial edema (circle), marked inflammatory cell infiltration (cube) and vacuolar degeneration of the cardiomyocytes (thin arrow), group III (HFD + 50 mg/kg of cichoriin) (**E**) showed normal cardiomyocytes (wave arrow), moderate vascular congestion (star), hemorrhage (arrowhead), few lipids deposition (thick arrow) and interstitial edema (circle), group IV (HFD + 100 mg/kg of cichoriin) (**F**) showed normal cardiac myocytes (wave arrow), mild congestion (star), hemorrhage (arrowhead) and less lipid deposition (thick arrow) and group V (10 mg/kg of atorvastatin) (**G**) showed mild to moderate vascular congestion (star), hemorrhage (arrowhead), normal cardiomyocytes (wave arrow) and interstitial edema (circle). High-fat diet (HFD). Scale bar, 50 μm.

The control group (Figure 2A) demonstrated the normal histological architecture of cardiomyocytes. They appear elongated, branched, and cross-striated with large oval central nuclei (the thick arrow) and narrow slit-like interstices (the arrowhead) in between. The HFD group (Figure 2B–D) showed severe histological alterations including severe vascular congestion (the star) and hemorrhage (the arrowhead) at the interstices between the cardiac myofibers, excessive lipid deposition (the thick arrow), distinct interstitial edema (the circle), and marked inflammatory cell infiltration (the cube) in addition to vacuolar degeneration of the cardiomyocytes (the thin arrow). The cichoriin-treated (50 mg/kg) group (Figure 2E) exhibited a moderate myocardial injury manifested by apparently normal cardiomyocytes (the wave arrow), moderate vascular congestion (the star), hemorrhage (the arrowhead), the few lipids deposition (the thick arrow), and interstitial edema (the circle). The cichoriin-treated (100 mg/kg) group (Figure 2F) revealed a marked improvement evidenced by nearly normal cardiac myocytes (the wave arrow), mild congestion (the star), and hemorrhage (the arrowhead), in addition to less lipid deposition (the thick arrow). The atorvastatin-treated group (Figure 2G) displayed moderate degenerative changes, including mild to moderate vascular congestion (the star) and hemorrhage (the arrowhead). Furthermore, the cardiomyocytes appeared nearly normal (the wave arrow), and the interstitial edema (the circle) was reduced to a great extent.

#### 3.5.2. Examination of Kidney Tissue

**Figure 3:**
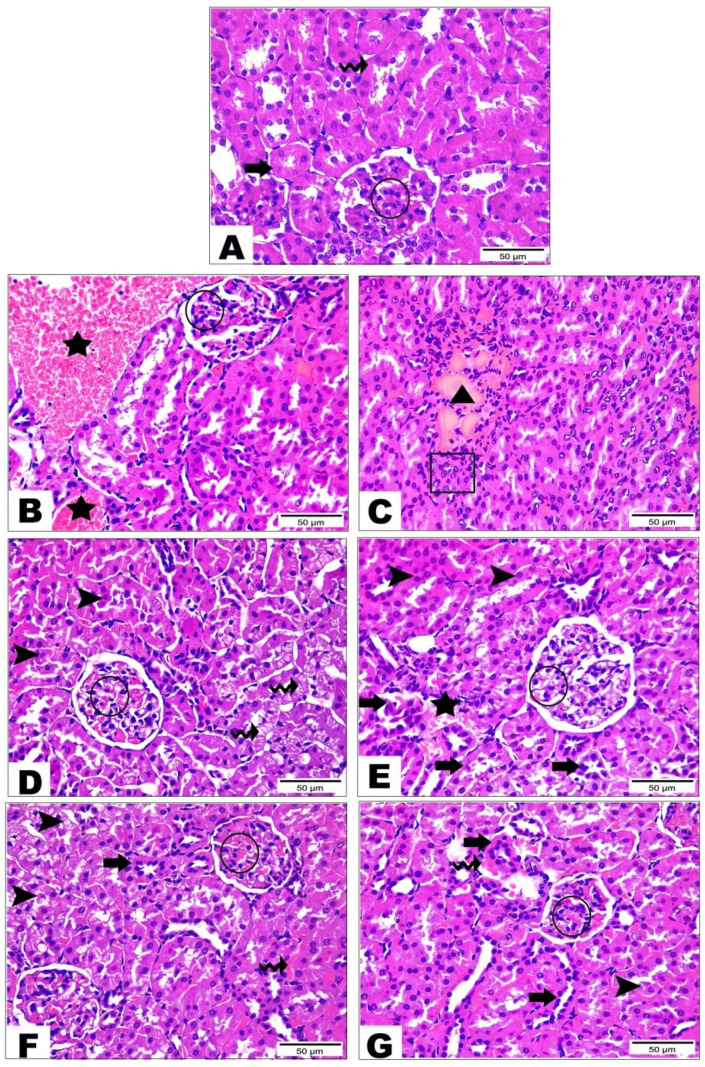
Photomicrograph sections of the control and HFD kidney tissue of rats with or without treatments. Group I (control) (**A**) showed the renal corpuscle (circle) and proximal (thick arrow) and distal (wave arrow) convoluted tubules, group II (HFD) (**B**–**D**) showed severe vascular congestion (black star), congestion of the renal corpuscle (circle), severe interstitial hemorrhage (triangle), inflammatory cells infiltration (thick arrow), degeneration with the desquamation of the epithelial lining (arrowheads) and cytoplasmic vacuolization with the pyknotic nuclei of the lining epithelium (wave arrow), group III (HFD + 50 mg/kg of cichoriin) (**E**) showed renal corpuscle with mild congestion (circle), renal tubules with an intact lining epithelium (arrowheads), mild vascular congestion (star) and a few renal tubules showing desquamated epithelial cells with pyknotic nuclei (thick arrows), group IV (HFD + 100 mg/kg of cichoriin) (**F**) and group V (10 mg/kg of atorvastatin) (**G**) showed tubular degeneration (arrowhead), pyknosis of the lining epithelium (thick arrow), mild hemorrhage interstitial between the renal tubules (wave arrow) and congestion inside the renal corpuscle (circle). High-fat diet (HFD). Scale bar, 50 μm.

The control group (Figure 3A) demonstrated the normal histological structure of the renal cortex containing the renal corpuscle (the circle) and proximal (the thick arrow) and distal (the wave arrow) convoluted tubules. The HFD group (Figure 3B–D) showed severe renal damage, including severe vascular congestion (the black star), as well as congestion of the renal corpuscle (the circle), severe interstitial hemorrhage (the triangle), and inflammatory cells infiltration (the thick arrow). Furthermore, some renal tubules displayed degeneration with the desquamation of the epithelial lining (the arrowheads), while others showed cytoplasmic vacuolization with the pyknotic nuclei of the lining epithelium (the wave arrow). The cichorin-treated (50 mg/kg) group (Figure 3E) exhibited a significant tissue recovery manifested by a nearly normal renal corpuscle with mild congestion (the circle) and renal tubules with an intact lining epithelium (the arrowheads). However, mild vascular congestion (the star) and a few renal tubules showing desquamated epithelial cells with pyknotic nuclei (the thick arrows) were still observed. The cichorin-treated (100 mg/kg) group (Figure 3F) and atorvastatin-treated group (Figure 3G) markedly decreased the renal damage observed in the positive control group. Both groups revealed a restoration of most of the histological structure of the renal cortex; however, tubular degeneration (the arrowhead) and pyknosis of the lining epithelium (the thick arrow), as well as mild hemorrhage interstitial between the renal tubules (the wave arrow) and congestion inside the renal corpuscle (circle) were still noticed but less so in severity than the positive control group.

#### 3.5.3. Examination of Liver Tissue

**Figure 4:**
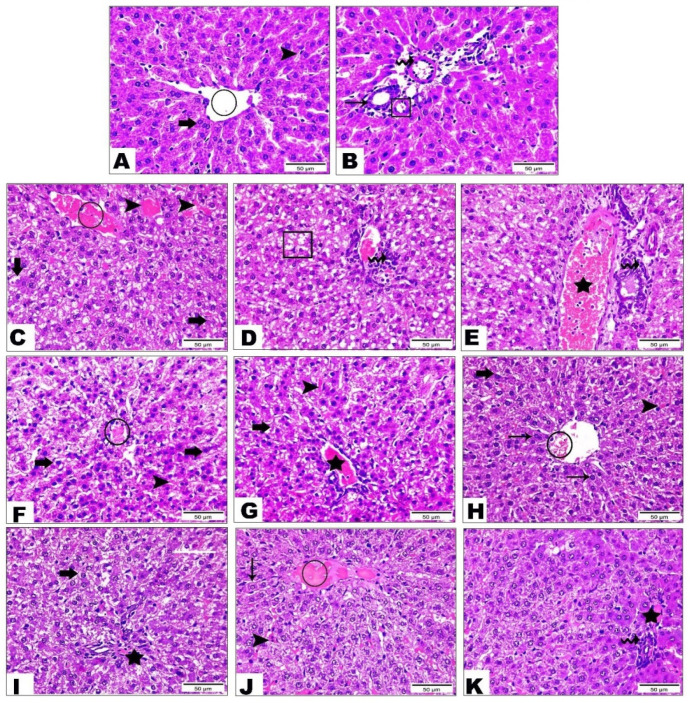
Photomicrograph sections of the control and HFD liver tissue of rats with or without treatments. Group I (control) (**A**,**B**) showed the hepatic central vein (circle), blood sinusoids (arrowhead), hepatic cords containing large hepatocytes (thick arrow), normal portal vein (wave arrow), hepatic artery (cube) and bile canaliculi (thin arrow), group II (HFD) (**C**–**E**) showed severe congestion of the central vein (circle), blood sinusoids (arrowhead), hepatocellular degeneration (arrow), micro-vesicular steatosis (cube), inflammatory cells infiltration (wave arrow)and severe congestion and dilatation of the portal vein (star), group III (HFD + 50 mg/kg of cichoriin) (**F**,**G**) showed mild congestion of the central vein (circle), blood sinusoids (arrowhead), moderate hepatocellular degeneration with the cytoplasmic vacuolation and pyknotic nuclei (arrow) and dilatation and congestion of the portal vein (star), group IV (HFD + 100 mg/kg of cichoriin) (**H**,**I**) showed a normal hepatic central vein (circle), regular hepatic cords with nearly normal hepatocytes (thin arrow), mild congestion of the blood sinusoids (arrowhead), portal vein (star) and mild hepatocellular degeneration (thick arrow) and group V (10 mg/kg of atorvastatin) (**J**,**K**) showed normal hepatocytes (thin arrow), congestion (arrowhead), congested hepatic central vein (circle), mild congestion of the portal vein (star), mild inflammatory cell infiltration (wave arrow). High-fat diet (HFD). Scale bar, 50 μm.

The control group (Figure 4A,B) showed the normal architecture of the central vein (the circle), blood sinusoids (the arrowhead), and hepatic cords containing large hepatocytes (the thick arrow) with central, spherical, and vesicular nuclei. The portal triad displayed a normal portal vein (the wave arrow), hepatic artery (the cube), and bile canaliculi (the thin arrow) (Figure 2B). The HFD group (Figure 4C–E) exhibited severe hepatic injury, including severe congestion of the central vein (the circle) and blood sinusoids (the arrowhead), hepatocellular degeneration (the arrow), micro-vesicular steatosis (the cube), inflammatory cells infiltration (the wave arrow), severe congestion, and dilatation of the portal vein (the star). The cichoriin-treated (50 mg/kg) group (Figure 4F,G) demonstrated a lesser hepatic injury than the positive control group, evidenced by the mild congestion of the central vein (the circle) and blood sinusoids (the arrowhead) and moderate hepatocellular degeneration with the cytoplasmic vacuolation and pyknotic nuclei (the arrow), as well as dilatation and congestion of the portal vein (the star). The cichoriin-treated (100 mg/kg) group (Figure 4H,I) revealed a marked improvement in the tissue architecture manifested by a normal central vein (the circle) and regular hepatic cords with nearly normal hepatocytes (the thin arrow) and portal area (Figure 4I). However, mild congestion of the blood sinusoids (the arrowhead) and portal vein (the star), in addition to mild hepatocellular degeneration (the thick arrow), were still noticed. The atorvastatin-treated group (Figure 4J,K) presented a remarkable recovery, evidenced by apparently normal hepatocytes (the thin arrow) with vesicular nuclei and prominent nucleoli, mild sinusoidal dilatation, and congestion (the arrowhead), whereas the central vein (the circle) showed severe congestion. The portal triad exhibited mild congestion of the portal vein (the star) with mild inflammatory cell infiltration (the wave arrow).

### 3.6. Effect of Cichoriin on Gene and Protein Expressions in Adipose Tissue

**Figure 5:**
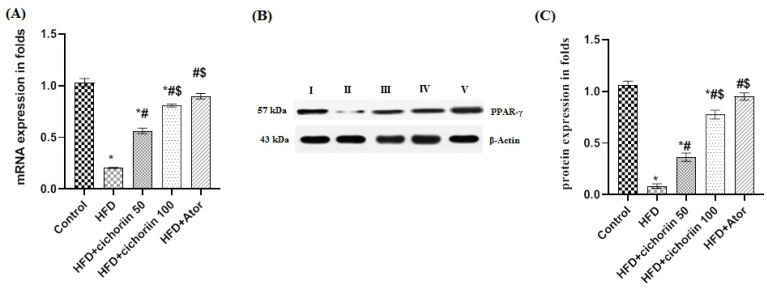
mRNA (**A**) and protein expressions (**B**,**C**) of PPAR-γ in adipose tissue of different groups. Data are presented as means ± SEM (n = 3). *, ^#^, and ^$^ indicate significant difference from the (control), (HFD), and (HFD + cichoriin 50 mg/kg) groups, respectively, at (*p* < 0.05). Group I (control); group II (HFD); group III (HFD + 50 mg/kg, cichoriin); group IV (HFD + 100 mg/kg, cichoriin); group V (10 mg/kg, atorvastatin); torvastatin (Ator); peroxisome proliferator-activated receptor gamma (PPAR-γ).

As shown in Figure 5, the PPAR-γ expression was significantly inhibited by the HFD in both the gene and protein levels compared to that of the control. The rats treated with cichoriin displayed a significant upregulation of the PPAR-γ expression in a dose-dependent manner. The high dose of cichoriin (100 mg/kg) was non-significantly different in the PPAR-γ expression from that of the atorvastatin group.

## 4. Discussion

Obesity is a growing worldwide problem of an accumulation of or excess of unhealthy body fats and is associated with various metabolic diseases. There is a positive correlation between daily fat intake and the incidence of obesity in humans, as well as in animals [45]. A plethora of herbs and phytocompounds demonstrated promising results in the prevention and treatment of obesity [4,46]. *Cichorium intybus* and its contents of coumarins are an antioxidant traditional herbal supplement with anti-diabetic and weight-reducing activities [47,48,49]. Hence, screening of coumarins and their derivatives as anti-obesity agents emphasized the importance of these molecules as lead compounds for developing potent drugs for the treatment of obesity and related disorders. In this study, the potential therapeutic effect of cichoriin, a coumarin derivative, in HFD-induced obesity was evaluated, as well as its ability to reduce the associated obesity complications on the liver, kidneys, and heart. Daily supplementation of cichoriin (50 or 100 mg/kg) for one month not only reduced the HFD-induced overweight, BMI, and visceral fat content but also improved the serum lipid profile in a dose-dependent manner, which is in concordance with the reported effect of the coumarins on the reduction of weight gain and abdominal fat mass in laboratory animals, as well as the total lipids and cholesterol levels [50]. Additionally, Cichoriin significantly reduced the elevated LDL-C/HDL-C ratio in the HFD rats, indicating the prevention of the risk of coronary vascular diseases associated with obesity [51]. Notably, a high dose of cichoriin was similar to the effect of atorvastatin, the commercially used statin drug, in the treatment of obesity.

Moreover, the cichoriin supplement significantly decreased the weight and lipid accumulation in the hepatic, renal, and cardiac tissues compared to the HFD-fed rats. It improved hepatic, cardiac, and renal dysfunction, as indicated by significantly lowered serum levels of ALT, AST, and CK-MB, as well as of urea and creatinine levels. Furthermore, steatosis and inflammatory cellular infiltrate, induced by the hepatic, cardiac, and renal lipotoxicity, were significantly inhibited by the cichoriin treatment. These findings are consistent with the reduction of serum and hepatic TG, TC, and LDL-C, as well as of the AST and ALT levels and the elevation of HDL-C by coumarin-rich *Grifola frondose* fungus ethanol extract administration in the high-fat diet rats [52] and the effect of coumarin derivatives (esculetin and coumarin dihydro-quinazolinone) [32,53,54,55].

Based on previous studies, HFD-induced obesity caused oxidative stress as the overproduction in lipid peroxidation and decreased antioxidant capacity [56,57]. Cichoriin ameliorated the oxidative stress induced by the HFD. As it decreased the lipid peroxidation product (MDA) content, it increased the antioxidant GSH level in the hepatic and renal tissues of HFD-induced obesity in rats. The administration of osthol (a coumarin derivative) prohibited kidney damage and renal lipotoxicity induced by a high-fat/high-sugar Western diet via preventing oxidative stress [58]. Therefore, the observed hepatorenal protective effect of cichoriin may be attributed to its antioxidant activity.

To understand the underlying molecular mechanism by which cichoriin impacts anti-obesity activity, the gene and protein expression of PPAR-γ in adipose tissues were measured. PPAR-γ is a nuclear receptor that regulates inflammation and the lipid/carbohydrate metabolism and, therefore, implicates therapeutic potential in various diseases, including obesity [59]. The results show that cichoriin upregulated PPAR-γ in both the mRNA and protein levels.

Numerous natural derivatives act as ligands of lipid-sensing nuclear receptors and regulate their activities [60]. Auraptene, a coumarin compound abundant in citrus, has been identified as a PPAR-γ agonist in adipocytes [61]. Subsequently, it reduces inflammation and fat mobilization in the liver [62]. Also, osthol activates PPAR-γ to modulate lipogenesis in oleic acid or HFD-induced-hepatic steatosis [63,64]. To our knowledge, this is the first study that has uncovered the molecular mechanism of the anti-obesity activity of cichoriin.

## 5. Conclusions

In the current study, cichoriin treatments suppressed the body-weight gain and serum lipids profile. Additionally, it restored the oxidative balance, structure, and function of the hepatic, as well as renal, tissues. Furthermore, cichoriin inhibited fat accumulation and lipotoxicity in cardiac tissue. The notable curative effect of cichoriin may be attributed to the upregulation of PPAR-γ. The results are that cichoriin is an interesting candidate that can be developed for the management of obesity and the improvement of its related metabolic complications. The study recommends further clinical studies on cichoriin to establish its possible application as a new strategy against obesity.

## Figures and Tables

**Table 1:** Composition of the prepared HFD.

Components	Amount (g for Total 1 kg Diet)
Normal pellet diet	365
Beef fat	310
Casein	250
Vitamins and minerals mixture	63
Cholesterol	10
Sodium chloride	1
Dried yeast	1

**Table 2:** Effect of cichoriin on body weight, BMI, and organ weight of obese rats.

Groups	Control	HFD	HFD + cichoriin 50 mg/kg	HFD + cichoriin 100 mg/kg	HFD + Ator 10 mg/kg
Body-weight gain (g)	106 ± 8	202 ± 8 *	142 ± 118 *^#^	123 ± 8 ^#^	145 ± 7 *^#^
BMI (g/cm^2^)	0.53 ± 0.01	0.71 ± 0.004 *	0.58 ± 0.02 *^#^	0.55 ± 0.002 ^#^	0.58 ± 0.01 *^#^
Liver (g)	6.8 ± 0.4	8.5 ± 0.4 *	7.0 ± 0.2 ^#^	6.7 ± 0.4 ^#^	6.8 ± 0.3 ^#^
Kidney (g)	1.3 ± 0.1	1.7 ± 0.1 *	1.5 ± 0.1	1.2 ± 0.04 ^#$^	1.4 ± 0.1 ^#^
Heart (g)	0.9 ± 0.04	1.2 ±0.1 *	1.1 ± 0.1	0.9 ± 0.04 ^#$^	1.0 ± 0.05
Visceral fat (g)	2.2 ± 0.3	7.1 ± 0.4 *	4.7 ± 0.7 *^#^	3.1 ± 0.2 ^#^	4.3 ± 0.5 *^#^

Data are presented as means ± SEM (n = 5). *, ^#^, and ^$^ indicate significant difference from the (control), (HFD), and (HFD + cichorin 50 mg/kg) groups, respectively, at (*p* < 0.05). High-fat diet (HFD); atorvastatin (Ator); body mass index (BMI).

**Table 3:** Effect of cichoriin on biochemical parameters in the rats.

Groups	Control	HFD	HFD + cichoriin 50 mg/kg	HFD + cichoriin 100 mg/kg	HFD + Ator 10 mg/kg
TC (mg/dL)	103 ± 9	168 ± 12 *	125 ± 6 ^#^	85 ± 5 ^#$^	86 ± 4 ^#$^
TG (mg/dL)	36 ± 2	68 ± 4 *	48 ± 1 *^#^	40 ± 2 ^#^	38 ± 2 ^#^
LDL-C (mg/dL)	69 ± 8	138 ± 12 *	93 ± 7 ^#^	44 ± 4 ^#$^	44 ± 4 ^#$^
HDL-C (mg/dl)	27 ± 1.5	16 ± 1.4 *	23 ± 1.4 ^#^	33 ± 1.8 ^#$^	34 ± 2.2 *^#$^
LDL-C/HDL-C	2.6 ± 0.2	8.9 ± 1.1 *	4.2 ± 0.5 ^#^	1.4 ± 0.1 ^#$^	1.3 ± 0.2 ^#$^

Data are presented as means ± SEM (n = 5). *, ^#^, and ^$^ indicate significant difference from the (control), (HFD), and (HFD + cichorin 50 mg/kg) groups, respectively, at (*p* < 0.05). High-fat diet (HFD); atorvastatin (Ator); total cholesterol (TC); triglycerides (TG); low-density lipoprotein cholesterol (LDL-C); high-density lipoprotein cholesterol (HDL-C).

**Table 4:** Effect of cichoriin on oxidative stress markers.

Groups	Control	HFD	HFD + cichoriin 50 mg/kg	HFD + cichoriin 100 mg/kg	HFD + Ator 10 mg/kg
Hepatic MDA (nmol/g tissue)	82.9 ± 3.6	136.5 ± 2.3 *	107.2 ± 1.8 *^#^	96.9 ± 3.4 *^#^	96.2± 3.7 *^#^
Renal MDA (nmol/g tissue)	67.0 ± 1.4	118.8 ± 2.6 *	78.4 ± 3.0 *^#^	64.3 ± 3.0 ^#$^	66.2 ± 1.9 ^#$^
Hepatic GSH (mg/g tissue)	634 ± 20	202 ± 9 *	271 ± 8 *^#^	325 ± 3 *^#$^	328 ± 6 *^#$^
Renal GSH (mg/g tissue)	556 ± 5	180 ± 13 *	286 ± 12 *^#^	339 ± 19 *^#$^	336 ± 6 *^#^

Data are presented as means ± SEM (n = 5). *, ^#^, and ^$^ indicate significant difference from the (control), (HFD), and (HFD + cichorin 50 mg/kg) groups, respectively, at (*p* < 0.05). High-fat diet (HFD); atorvastatin (Ator); malondialdehyde (MDA); reduced glutathione (GSH).

## Data Availability

The data presented in this study are available on request from the corresponding author.
